# Luteolin attenuates CCl4-induced hepatic injury by inhibiting ferroptosis via SLC7A11

**DOI:** 10.1186/s12906-024-04486-2

**Published:** 2024-05-16

**Authors:** Zhiqiang Han, Batudeligen, Hongmei Chen, Narisu, Anda, Yanhua Xu, Lan Xue

**Affiliations:** Institute of Clinical Pharmacology of Traditional Mongolian Medicine, Affiliated Hospital of Inner Mongolia Minzu University, No.1742, Huolinhe Street, Horqin Area, Tongliao City, Autonomous Region of Inner Mongolia 028000 China

**Keywords:** Hepatic injury, Luteolin, Ferroptosis, SLC7A11, GPX4

## Abstract

**Background:**

Luteolin (3,4,5,7-tetrahydroxy flavone) is reported to strongly protect from acute carbon tetrachloride (CCl_4_) -induced liver injury or fibrosis. Ferroptosis can be induced by hepatic injury, and contributes to liver fibrosis development. The exact functional mechanism underlying luteolin inhibition of hepatic injury and whether ferroptosis is involved are unclear.

**Methods:**

Mice model and cell model of liver injury were constructed or induced to explore the effect and molecular mechanisms of Luteolin in the treatment of hepatic injury using CCl4. Cell Counting Kit-8 (CCK-8) and flow cytometry were used to evaluate HepG2 cell viability and apoptosis. The differential expressed genes involved in liver injury were scanned using RNA-seq and confirmed using functional study. Western blot was used to detect the indicators related to ferroptosis.

**Results:**

Luteolin attenuated hepatic injury by alleviating cell morphology and decreasing serum aspartate aminotransferase (AST), alanine aminotransferase (ALT), and alkaline phosphatase (ALP) levels in vivo mice models, and increasing cell viability, downregulating arachidonate 12-lipoxygenase (ALOX12), cyclooxygenase-2 (COX-2) and P21 protein expression, suppressing apoptosis in vitro cell models. Luteolin also inhibited ferroptosis by stimulating glutathione peroxidase 4 (GPX4) and mitochondrial ferritin (FTMT) protein expression, increasing glutathione (GSH) content, and minimizing Fe^2+^ and malondialdehyde (MDA) levels. Solute carrier family 7a member 11 (SLC7A11) was identified to be a key regulatory gene that participated in luteolin attenuation of CCl_4_-induced hepatic injuries in HepG2 cells using Microarray assay. Functional study showed that SLC7A11 can alleviate hepatic injury and ferroptosis.

**Conclusion:**

Luteolin attenuated CCl_4_-induced hepatic injury by inhibiting ferroptosis via SLC7A11. SLC7A11 may serve as a novel alternative therapeutic target for hepatic injury.

**Supplementary Information:**

The online version contains supplementary material available at 10.1186/s12906-024-04486-2.

## Background

Hepatic injury is a relatively common occurrence, due to the anatomic location and size of the liver [1]. Due to genetic and environmental risk factors, drugs can be harmful to the liver in susceptible individuals by causing cellular stress, cell death, activation of an adaptive immune response and a failure to adapt, with progression to overt liver injury [2]. Carbon tetrachloride (CCl_4_) is a strong nephrotoxic, hepatoxic, and prooxidant agent, that is widely applied to induce hepatotoxicity in experimental animals and has been used in recent research to create hepatic fibrosis or cirrhosis, hepatocellular carcinoma, liver injury, and chemical hepatitis models [3]. In a matter of dose, exposure time of CCl_4_, or age of the affected organism, regeneration can take place and lead to full recovery from liver damage [4]. Lipid-peroxidation-induced oxidative changes may be the mechanism underlying the damage-causing mechanism of CCl_4_ in tissues, and antioxidants from plants are promising therapeutic drugs against hepatic injury [3].

Luteolin (3,4,5,7-tetrahydroxy flavone) is a common flavonoid found in plants, including fruits, vegetables, and herbal medicines [5]. Luteolin is reported to have significant protective effects against acute liver injury and liver fibrosis induced by CCl_4_, lipopolysaccharide (LPS), and D-galactosamine/LPS [6–10]. The mechanism was related to promoting extracellular matrix degradation in the fibrotic liver tissue and the strong enhancement of hepatic regenerative capability [6], inhibition of the extrinsic and intrinsic apoptotic pathways [10], inhibiting Thioredoxin Interacting Protein (TXNIP) - NLR Family Pyrin Domain Containing 3 (NLRP3) inflammasome [8], regulating the release of HMGB1 through the P2 × 7R-RAGE-TLR4 axis [9] and targeting AKT/mTOR/p70S6K and TGFβ/Smad signaling pathways [7].

Ferroptosis, is a novel form of programmed cell death that acts in an iron-dependent manner and is characterized by substantial accumulation of lipids peroxides, as well as an imbalanced cell redox state, and is essential in regulation of liver disease occurrence and development [11, 12]. Ferroptosis regulatory pathways can be broadly classified into three groups: the glutathione/glutathione peroxidase 4 (GSH/GPX4), iron metabolism, and lipid metabolism pathways [12, 13], and ferroptosis could induce liver fibrosis [14]. Sorafenib attenuates liver fibrosis by triggering hepatic stellate cell ferroptosis via HIF-1α/SLC7A11 pathway [15]. HBV X protein potentiated D-GalN-induced hepatotoxicity and ferroptosis in vitro, and it suppressed SLC7A11 expression through H3K27me3 modification by EZH2 [16]. However, the regulatory mechanism of ferroptosis in Luteolin needs further investigation.

The aim of this study is to investigate the potential underlying mechanisms by which luteolin attenuates hepatic injury through ferroptosis signaling which may open the possibility to treat liver injury with drugs that inhibit ferroptosis.

## Materials and methods

### Cells and reagents

Human HepG2 cells were bought from the Cell Bank of Shanghai Institute of Cell Biology, Chinese Academy of Sciences, and cultivated in DMEM (Gibco, Carlsbad, CA, USA) supplemented with 1% penicillin/streptomycin solution (Hyclone, Cat: SV30010) and 10% fetal bovine serum (Gibco) in a humidified environment comprising 5% CO_2_ and 95% air at 37 °C.

Luteolin and CCl_4_ were acquired from Sigma-Aldrich (Saint Louis, MO, USA). Erastin was from Selleck (S7242, Selleck China). Commercial kits for measuring serum alanine aminotransferase (ALT, #C009-1-1), aspartate aminotransferase (AST, #C010-2-1), and alkaline phosphatase (ALP, #A059-1-1) were from Jiancheng Bioengineering Institute (Nanjing, China) and were analyzed using a fully automatic biochemical analyzer (BS-240VET, Mindray Bio-medical Electronics Company, Ltd, Shenzhen, China). Antibody against p21 was from Abcam, and antibodies against arachidonate 12-lipoxygenase (ALOX12), cyclooxygenase-2 (COX-2), glutathione peroxidase 4 (GPX4), mitochondrial ferritin (FTMT), heme oxygenase 1 (HO-1), solute carrier family 7a member 11 (SLC7A11), and GAPDH were all from Invitrogen.

### Animal experiments

Male C57BL/6 mice were from Beijing Vital River Animal Experimental Technology (Beijing, China). Mice were randomly allocated to Vehicle group (olive oil) and CCl_4_ group (treated with 0.2 mg/kg). Then, each of the above two groups were further divided into two groups with Vehicle (0.5% CMC-Na) and luteolin (40 mg/kg). Treatment of mice with CCl_4_ was conducted by intraperitoneal (i.p.) injection, twice a week for six consecutive weeks. After 4 weeks of CCL_4_ treatment, luteolin was administered i.g. once a day for two consecutive weeks at doses of 40 mg/kg of body weight. The health of mice was carefully monitored daily until the end of the study. After 24 h of the last treatment, all mice were injected intraperitoneally sodium pentobarbital (Sigma Aldrich) for euthanasia, followed by collection of tissue and serum samples; blood samples were immediately obtained from the abdominal aorta, then stored at -80 °C for biochemical analysis. Liver tissue samples were rinsed twice with 50 mM Tris-HCl (neutral pH), then fixed in 10% buffered formalin for histopathological examination. The experiments were approved by the Ethics Committee of Affiliated Hospital of Inner Mongolia Minzu University. The animal study is reported in accordance with ARRIVE guidelines (https://arriveguidelines.org).

### Histopathological examination

After dehydration and paraffinization of liver tissue specimens fixed in 4% paraformaldehyde (PFA) for 12 h, Sect. (5-µm thickness) were cut and stained with hematoxylin and eosin (H&E) or Masson’s staining for light microscopy examination. A Nikon microscope (Eclipse E200-LED, Tokyo, Japan; magnification power, 400×) was used to observe specimens.

### Cell viability determination

HepG2 cells in logarithmic phase growth were plated into 96-well plates (density, 2 × 10^4^ cells/well) in 100 µL medium per well. Cells were treated with vehicle (DMSO), CCl4 (3.5mM), CCl4 (3.5mM) + luteolin (5 µM), and CCl4 (3.5mM) + luteolin (5 µM) + erastin (2.5 µM) according to the experimental design. After culture for 24 h, cell viability was evaluated in each group by CCK-8 assay. The effects of luteolin on cell viability were assessed by treating HepG2 cells with 0, 2.5, 5, 10, 20, 30, 40, 80 and 100 µM luteolin, and culture for 48 h, followed by CCK-8 assay. To assess the effects of SLC7A11 on cell viability, HepG2 cells were transfected with siRNAs or overexpression plasmids and cultivated for 24, 48, 72, and 96 h, followed by CCK-8 assay.

### Western blot

HepG2 cells were lysed in lysis buffer (Beyotime, China) containing protease inhibitor. Protein concentration was determined using bicinchoninic acid protein assay kits (Beyotime, China). 20 µg of proteins were loaded on to 10% SDS-PAGE gels for separation, then transferred onto polyvinylidene fluoride membranes. Membranes were blocked using 5% nonfat milk for ≥ 1 h, then incubated overnight at 4 °C with primary antibodies against ALOX12 (ab168384, abcam, 1:1000), COX2 (ab188184, abcam, 1:1000), p21 (ab109199, abcam, 1:1000), GPX4 (ab116703, abcam, 1:1000), FTMT (ab124889, abcam, 1:1000), HO-1 (ab13243, abcam, 1:1000), SLC7A11 (ab175186, abcam, 1:1000) and GAPDH (ab181602, abcam, 1:1000), and then rinsed three times with phosphate buffered saline (PBS) containing Tween (PBST), following by incubation with anti-rabbit or anti-mouse secondary antibodies for 1 h at 37 °C. Next, membranes were rinsed three times in PBST, and an Odyssey two-color infrared laser imaging system (LI-COR Biosciences, Lincoln, NE, USA) used to visualize protein expression, with ImageJ analysis software applied to quantify grayscale values.

### Apoptosis assay

HepG2 cells were seeded in 6-well culture plates at 1 × 10^7^ well/ml, then treated with reagents, according to the experimental design. After incubation for 48 h, cells were harvested by EDTA-free trypsinization to prepare single cell suspensions, followed by centrifugation at 1000 ×g for 5 min and discarding the supernatant. Cells were resuspended in approximately 1 ml PBS, precooled to 4 °C. Cells were pelleted by centrifugation and apoptosis detected using an Annexin V - FITC/PI apoptosis double staining kit (Absin, cat: abs50001) on a Millipore flow cytometer (Guava easyCyte HT, France).

### Determination of intracellular GSH, Fe^2+^, and malondialdehyde (MDA) levels

HepG2 cells were seeded in 6-well plates (1 × 10^5^ cells/well), then cultivated for 24 h in culture medium. After treatment with various reagents or transfection with siRNAs or plasmids, HepG2 cells were rinsed three times with ice-cold PBS, extracted using 5% trichloroacetic acid, and then treated with ether to remove the trichloroacetic acid. Total glutathione content in the aqueous layer was measured using an enzymatic method, based on the catalytic action of glutathione in the reduction of 5,5′-dithiobis (2-nitrobenzoic acid) by the glutathione reductase system [17]. For iron detection, harvested cells were immediately homogenized in PBS. Following centrifugation, Fe^2+^ level was detected in the supernatant using an Iron Assay Kit (Abcam, Shanghai, China), according to the manufacturer’s instructions. MDA levels were determined using a commercially available assay kit (Beyotime, Institute of Biotechnology, Haimen, China), which was calibrated by protein content, and the protein determination was measured by BCA method.

### Microarray assay

The total RNA of three groups of HegG2 cells, Vehicle, CCl_4_ treatment, CCl_4_ + luteolin treatment, were exacted by TRizol reagent and then depurated with RNeasy mini spin columns. The samples were quantitative by NanoDrop ND-1000 spectrophotometer. And the microarray assay was performed with the Affymetrix human Gene Chip primeview to determine the gene expression profile according to the manuscript. Significantly different genes were identified based on the criteria: the *P* < 0.05 and absolute fold change > 1.5.

### siRNA and plasmid transfection protocol

HepG2 cells were seeded into six-well plates at 2.5 × 10^4^ cells/well, Cells were maintained at 37ºC in RPMI-1640 medium containing 10% fetal bovine serum (FBS; GIBCO, Carlsbad, CA) in a humidified atmosphere containing 5% CO_2_. Small interfering RNAs (siRNAs) targeting SLC7A11 (siSLC7A11#1, 5’- CCUGUCACUAUUUGGAGCUUU − 3’; siSLC7A11#2, 5’-CCUGCGUAUUAUCUCUUUAUU-3’ siCtrl, 5’-UUCUCCGAACGUGUCACGU-3’) were designed and synthesized by Hippobio (Huzhou, China) for gene knockdown. Short interfering RNAs (siRNAs) for SLC7A11 and negative control RNA were constructed by Huzhou Hema Biological Technology Co., Ltd. The used primer pairs are shown in Supplementary Table. pcDNA3.1- SLC7A11 and corresponding control plasmids were all purchased from Beijing Tianyi Huiyuan Biotechnology Co., Ltd. (Beijing, China). Lentivirus packaging was conducted by transiently transfecting 293T cells with Lipofectamine 2000 reagent (Invitrogen).

### Reverse transcription-quantitative polymerase chain reaction (RT-qPCR)

Total RNA samples were extracted from HepG2 cells using TRIzol® reagent (Invitrogen, Carlsbad, CA, USA) according to the manufacturer’s instructions for RT-qPCR analysis of *SLC7A11* expression. Then, cDNA was reverse transcribed from an aliquot of extracted total RNA. RT-qPCR was performed using Power SYBR® Green PCR Master Mix (Applied Biosystems, Life Technologies, Foster, CA, USA) on a 7500 Real Time PCR System (Applied Biosystems, Foster City, CA, USA). The primer sequences used were shown in the Supplementary Table 1. The thermocycling conditions as follow: initial denaturation: 94℃ for 10 min, followed by 45 of cycles of denaturation: 94℃ for 30 s, annealing at 60℃ for 30 s, and extension at 72℃ for 30 s. β-actin was utilized for normalization, and the calculation of relative gene expression degrees was made with the 2^−∆∆Ct^ method.

### Statistical analysis

Data analyses were conducted using SPSS 18.0 (SPSS, Inc., Chicago, IL, USA). Quantitative data are presented as mean ± standard error of the mean, and the student’s t test was applied to evaluate the significance of differences between two groups. For comparisons among multiple groups, ANOVA was performed followed by Dunnett’s post-test. *P* < 0.05 was the threshold for statistical significance.

## Results

### Luteolin attenuates hepatic injury and liver fibrosis induced by CCl_4_ in mice

**Fig. 1:**
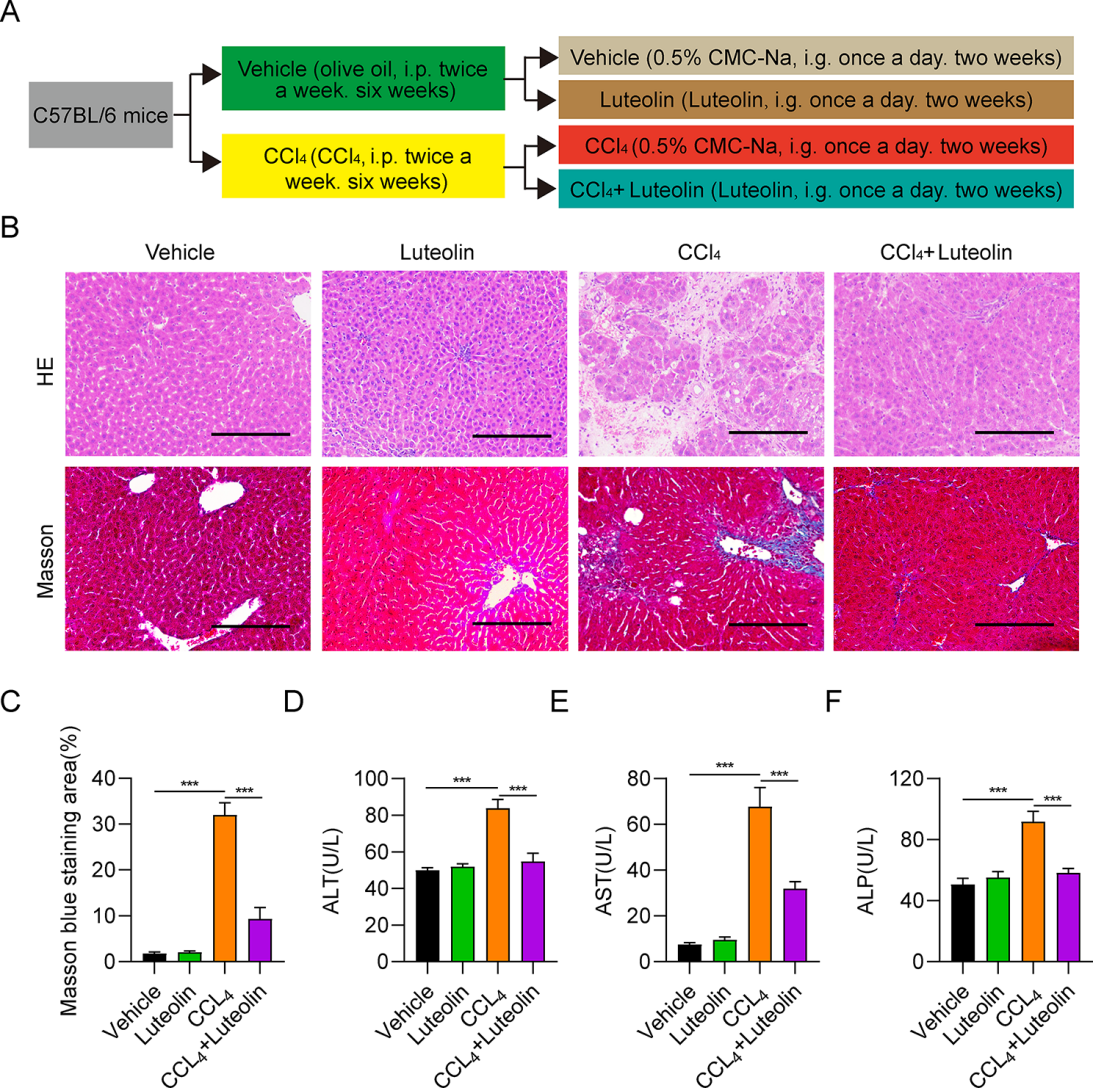
Luteolin attenuates CCl_4_-induced hepatic injuries and liver fibrosis in mice model. (**A**) Establishment of Luteolin attenuates CCl_4_-induced hepatic injuries and liver fibrosis in mice model. (**B, C**) Representative images and statistical analysis of H&E and Masson staining for comparison of liver tissue pathological changes among mice in vehicle, luteolin, CCl_4_, and CCl_4_ + luteolin groups. (**D**-**F**) Comparison of serum ALT, AST, and ALP content among mice in the vehicle, CCl_4_, and CCl_4_ + luteolin group. ^***^P < 0.001

CCl_4_ is a compound that can chemically induce hepatic injuries [18]. Establishment of Luteolin attenuates CCl_4_-induced hepatic injuries and liver fibrosis in mice model was shown in Fig. 1A. Our results showed that the tissue structure of mouse liver samples and cell morphology in vehicle and luteolin groups were normal, whereas liver samples from the CCl_4_ group were abnormal, with inflammatory cell infiltration and liver cell degeneration. Conversely, these abnormities were reversed in the liver of luteolin treatment, compared with the CCl_4_ groups. Furthermore, the masson’ s staining indicated that CCl_4_ markedly increased the formation of perivascular and interstitial fibrosis, while luteolin alleviated these phenotypes (Fig. 1B and C). ALT and AST are useful biomarkers of liver injury related to hepatic cellular integrity [19]. After injection of CCl_4_, ALT, AST, and ALP were significantly higher in the CCl_4_ group than in the vehicle group (Fig. 1D-F); however, relative to the CCl_4_ group, treatment with luteolin (40 mg/kg) markedly reduced ALT, AST, and ALP content (Fig. 1D-F). These results demonstrate that luteolin can attenuate hepatic injury caused by CCl_4_ in mice.

### Luteolin attenuates hepatic injury induced by CCl_4_ in hepatic cells

**Fig. 2:**
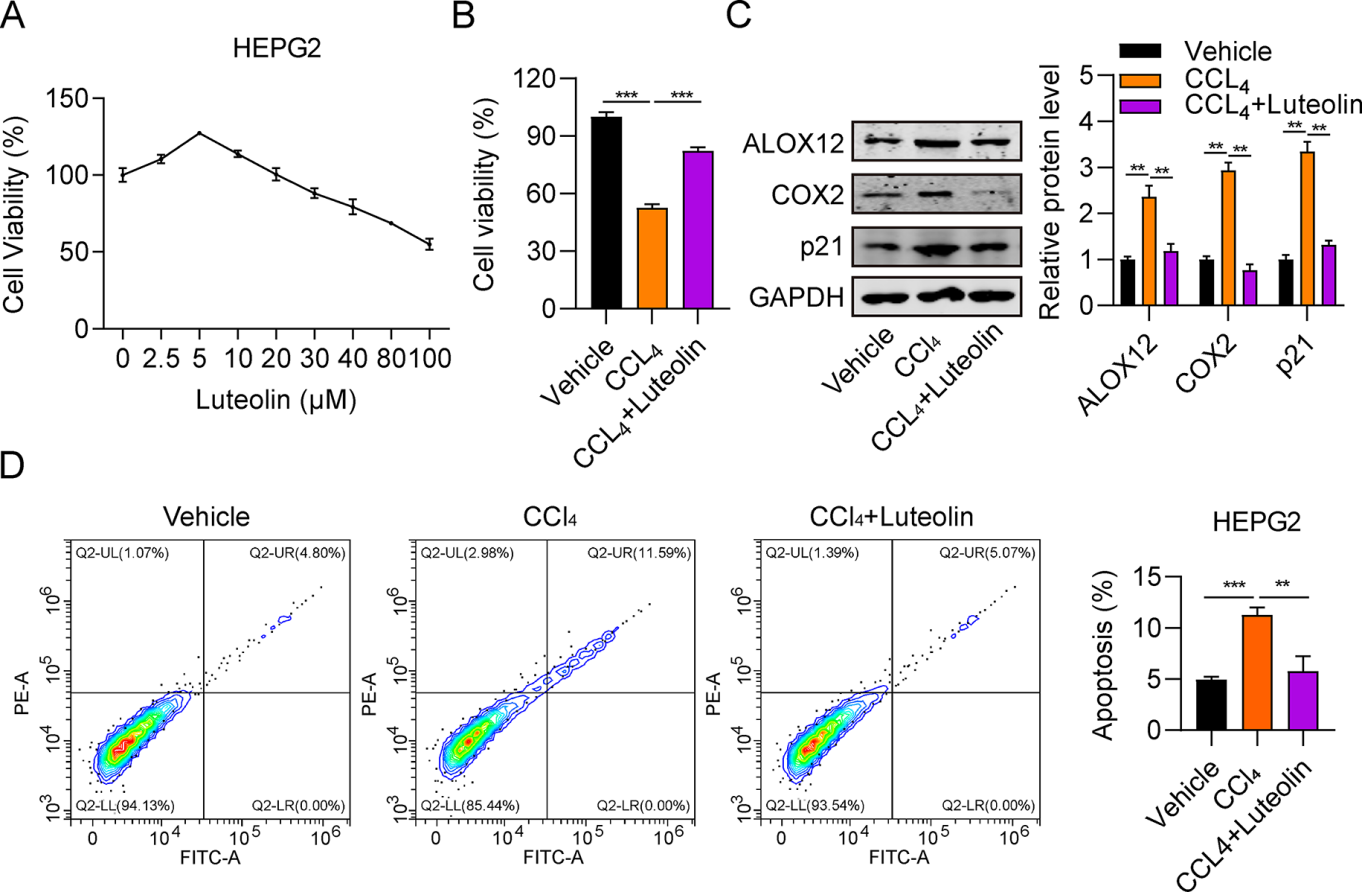
Luteolin attenuates CCl_4_-induced hepatic cell injury. (**A**) Viability of HepG2 cells treated with different concentrations of luteolin (2.5–100 µM) detected by CCK-8 assay. (**B**) Comparison of HepG2 cell viability among the vehicle, CCl_4_, and CCl_4_ + luteolin group, measured by CCK-8 assay. (**C**) Comparison of ALOX12, COX-2, and p21 protein levels in HepG2 cells in the vehicle, CCl_4_, and CCl_4_ + luteolin group, determined by western blot. (**D**) Comparison of HepG2 cell apoptosis rates among the vehicle, CCl_4_, and CCl_4_ + luteolin group, measured by flow cytometry. ^**^P < 0.01, ^***^P < 0.001

To explore the potential impact of luteolin on cell viability, HepG2 cells were treated with a series of luteolin concentrations (2.5–100 µM) for 48 h. As detected by CCK-8 assay, cell viability was slightly increased in cells treated with 5 µM luteolin, then decreased in the high concentration of luteolin (Fig. 2A), indicating that luteolin can promote cell viability; therefore, 5 µM luteolin was selected for use in further investigations. To assess the effect of luteolin on hepatic injury, HepG2 cells were treated with CCl_4_, or CCl_4_ + luteolin. The results of CCK-8 assays showed that HepG2 cell viability in the CCl_4_ group was significantly lower than that in the vehicle group, while viability in the CCl_4_ + luteolin group was greatly increased, relative to that in the CCl_4_ group (Fig. 2B).

To assess the degree of hepatic injury, we determined expression levels of three proteins (ALOX12, COX-2 and p21) associated with hepatic injury in HepG2 cells treated with CCl_4_ or CCl_4_ + luteolin by western blot analysis. After treatment with CCl_4_, ALOX12, COX-2, and p21 protein levels were all significantly increased (Fig. 2C), indicating that hepatic injury was induced. More importantly, levels of these proteins were all decreased in the CCl_4_ + luteolin group (Fig. 2C).

Apoptosis is a major feature of the pathogenesis of liver disease [20]. Flow cytometry was applied to assess apoptosis of HepG2 cells treated with CCl_4_ or CCl_4_ + luteolin. The apoptosis rate in the CCl_4_ group was significantly higher than that in the vehicle group, while that of CCl_4_ + luteolin group was greatly reduced relative to the CCl_4_ group (Fig. 2D); there was no significant difference between the vehicle and CCl_4_ + luteolin groups. These data demonstrate that luteolin can inhibit HepG2 cell apoptosis, indicating that luteolin can attenuate CCl_4_-induced hepatic cell injury.

### Luteolin inhibits hepatic cell ferroptosis

**Fig. 3:**
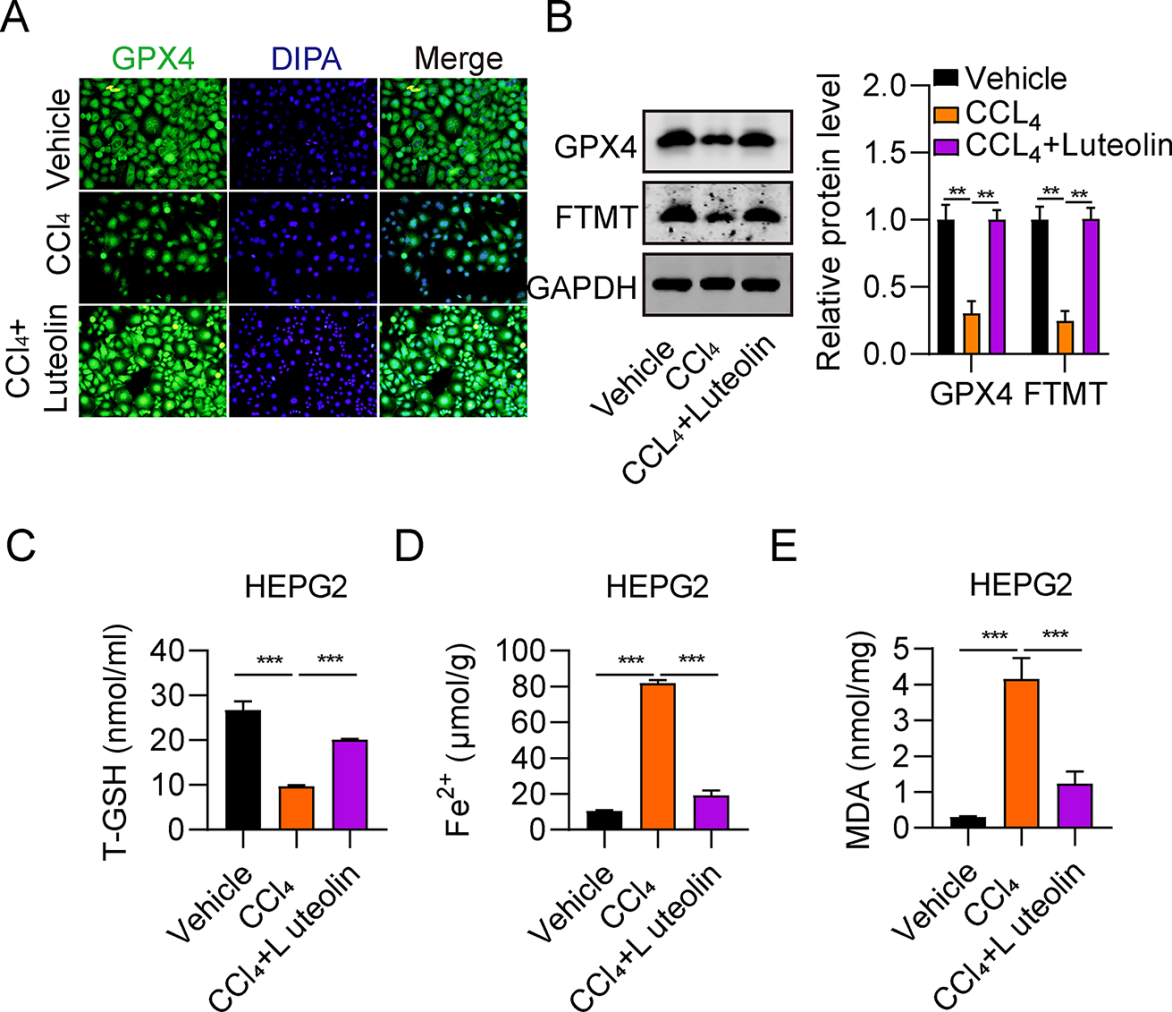
Luteolin inhibits hepatic cell ferroptosis. (**A**) Comparison of GPX4 levels in HepG2 cells among vehicle, CCl_4_, and CCl_4_ + luteolin group, evaluated by immunofluorescence staining. (**B**) Comparison of GPX4 and FTMT protein levels among HepG2 cells in the vehicle, CCl_4_, and CCl_4_ + luteolin group, determined by western blot. (**C**-**E**) Comparison of T-GSH, Fe^2+^, and MDA content in HepG2 cells among vehicle, CCl_4_, and CCl_4_ + luteolin group, measured using corresponding kits. ^***^P < 0.001

Next, we investigated the influence of luteolin on HepG2 cell ferroptosis in the vehicle, CCl_4_, and CCl_4_ + luteolin group. GPX4 is one of the most important antioxidant enzymes in mammals [21], the loss of lipid peroxide repair activity by glutathione peroxidase 4 (GPX4) is a distinct fingerprint of ferroptosis, and ferroptosis contribute to acute or chronic liver diseases [12]. GPX4 was markedly repressed in HepG2 cells according to immunofluorescence staining (Fig. 3A). FTMT, which functions as a cellular iron store, is a regulator of ferroptosis [22]. Here, we found that GPX4 and FTMT protein levels were markedly decreased in the CCl_4_ group relative to the vehicle group (Fig. 3B). Further, levels of GPX4 and FTMT proteins in the CCl_4_ + luteolin group were more substantially increased, relative to those in the CCl_4_ group (Fig. 3B). Moreover, a clear decrease of T-GSH level and apparent increases of Fe^2+^ and MDA levels were observed in the CCl_4_ group relative to the vehicle group (Fig. 3C-E). In summary, luteolin treatment increased T-GSH production in HepG2 cells, while it downregulated Fe^2+^ and MDA levels (Fig. 3C-E). These results demonstrate that luteolin inhibits ferroptosis in hepatic cells.

### Luteolin attenuates hepatic injury by inhibiting ferroptosis

**Fig. 4:**
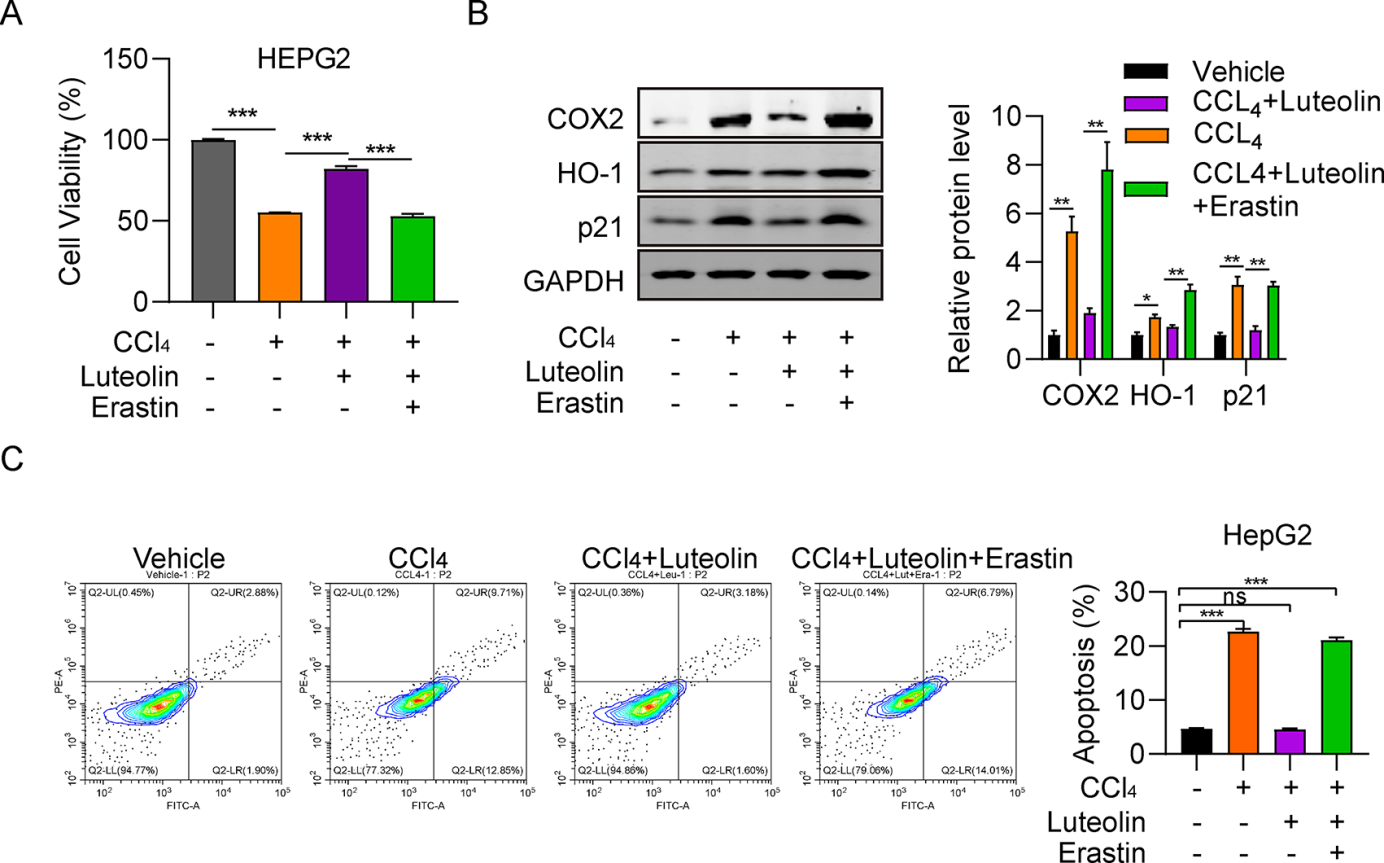
Luteolin attenuates hepatic injury by inhibiting ferroptosis. (**A**) Comparison of HepG2 cell viability among vehicle, CCl_4_, CCl_4_ + luteolin, and CCl_4_ + luteolin + erastin groups measured by CCK-8 assay. (**B**) Comparison of COX-2, HO-1, and p21 protein levels among HepG2 cells in vehicle, CCl_4_, CCl_4_ + luteolin, CCl_4_ + luteolin + erastin groups, determined by western blot. (**C**) Comparison of HepG2 cell apoptosis rates among vehicle, CCl_4_, CCl4 + luteolin, and CCl_4_ + luteolin + erastin groups, assessed by flow cytometry. ^***^P < 0.001, ns: not significant

Based on our findings that luteolin can influence both hepatic injury and ferroptosis, we next evaluated whether there was a connection between these two phenomena, using an inducer of ferroptosis, erastin, which initiates ferroptosis by disturbing cellular cystine uptake [23]. As shown in Fig. 4A, HepG2 cell viability was significantly increased by luteolin, and decreased substantially in response to treatment with erastin (2.5 µM), suggesting that the increase in HepG2 cell viability induced by luteolin can be reversed by ferroptosis induction. COX-2, HO-1, and p21 protein expression in the CCl_4_ + luteolin group were significantly lower than those in the CCl_4_ group, while they were higher in the CCl_4_ + luteolin + erastin group (Fig. 4B). Fluorescence-activated cell sorting analysis of HepG2 cells indicated that inhibition of apoptosis by luteolin can be ameliorated by erastin (Fig. 4C), suggesting that luteolin attenuates hepatic injury by inhibiting ferroptosis.

### Key genes involve in luteolin attenuation of hepatic injury

**Fig. 5:**
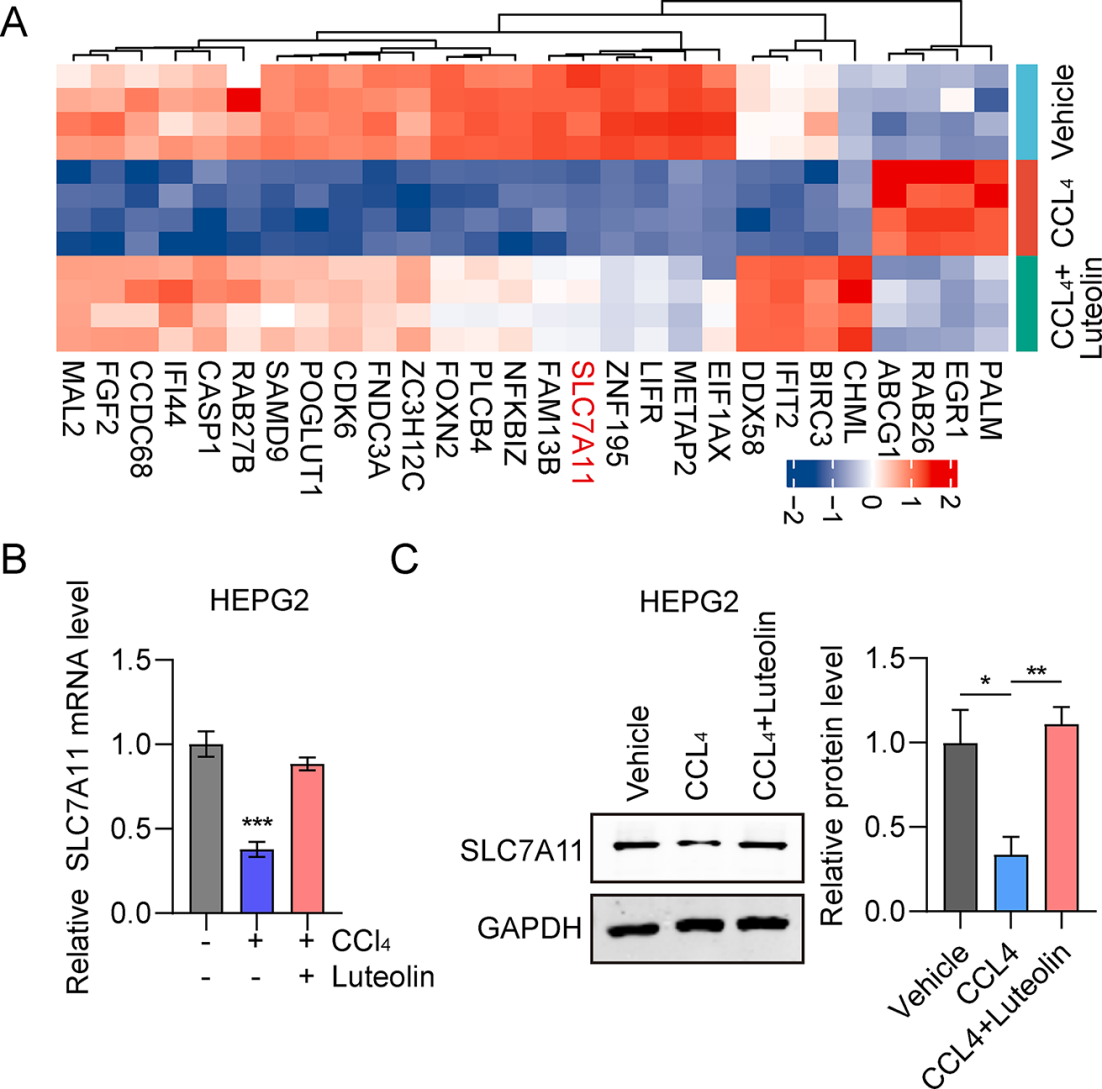
Key genes involve in luteolin attenuation of hepatic injury. (**A**) The top 30 genes involved in luteolin attenuation of hepatic injury were screened using RNA-seq. (**B**, **C**) Comparison of SLC7A11 mRNA and protein levels among HepG2 cells in vehicle, CCl_4_, and CCl_4_ + luteolin group. ^***^P < 0.001

Given the association among the luteolin, hepatic injury, and ferroptosis, we next explored the underlying mechanism. Microarray was used to screen for key genes involved in luteolin attenuation of hepatic injury of HepG2 cells in the vehicle, CCl_4_, and CCl_4_ + luteolin groups. We found four genes (EGR1, PALM, RAB26 and ABCG1) were upregulated in CCl_4_ treatment group, while downregulated in luteolin treatment group. Furthermore, 24 genes (SLC7A11, CASP1, FGF2, CDK6, METAP2, NFKBIZ, LIFR, IFIT2, FNDC3A, CCDC68, POGLUT1, ZC3H12C, CHML, IFI44, MAL2, PLCB4, BIRC3, EIF1AX, DDX58, FAM13B, FOXN2, SAMD9 ZNF195 and RAB27B) were downregulated in CCl4 group treatment group, while upregulated in luteolin treatment group (Fig. 5A). It has been reported that SLC7A11 acted as a crucial component of a cystine/glutamate antiporter, critical for maintaining intracellular GSH homeostasis and inhibited ferroptosis [24]. To confirm the microarray results, we detected the expression of SLC7A11 in CCl_4_ or CCl_4_ + luteolin treatment by RT-qPCR and western blot assay. The results showed that both the mRNA and the protein level were downregulated in CCl_4_ treatment, while upregulated in CCl_4_ + luteolin treatment (Fig. 5B and C).

### Effect of SLC7A11 on hepatic injury and ferroptosis

**Fig. 6:**
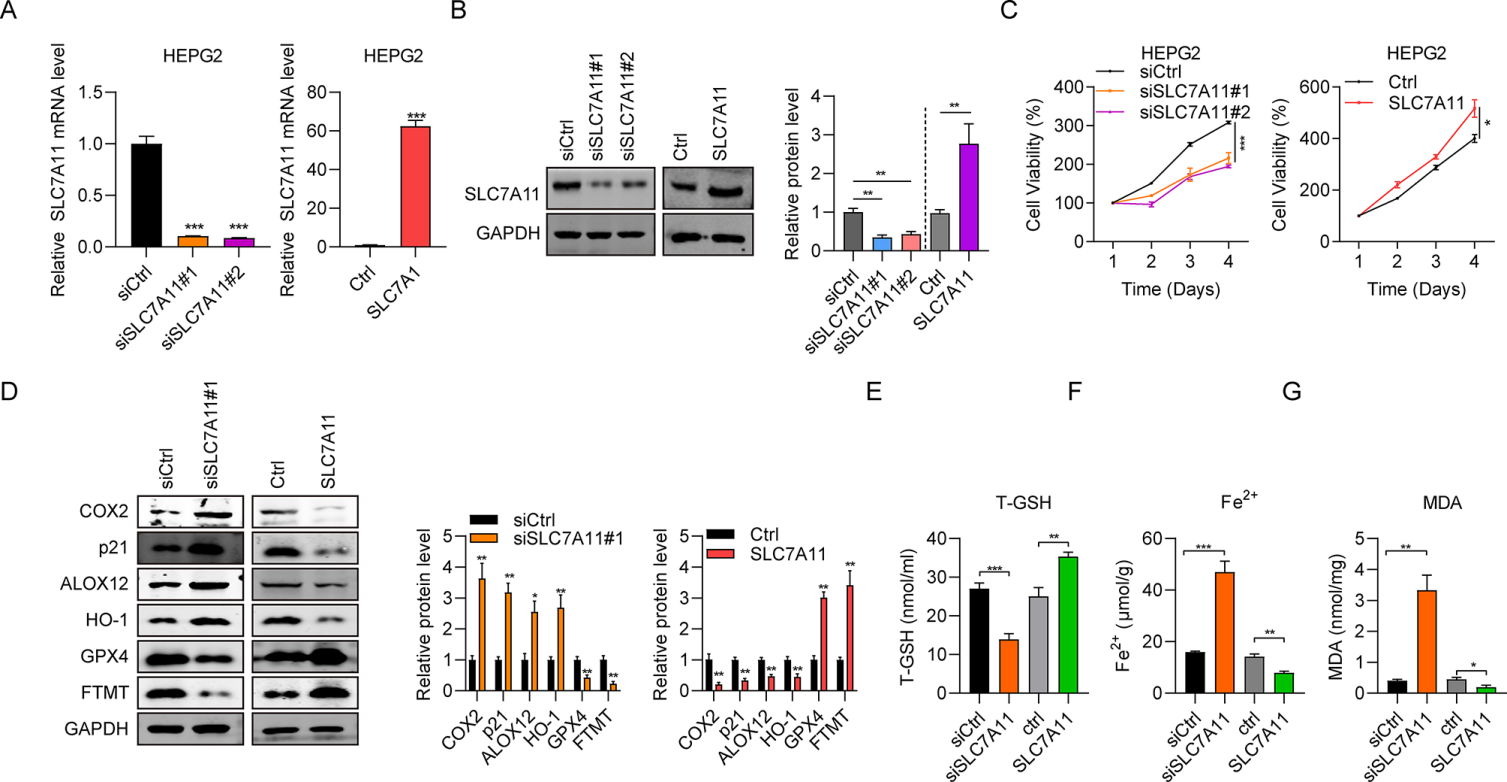
The influence of SLC7A11 on hepatic injury and ferroptosis. (**A**, **B**) SLC7A11 mRNA expression and protein expression in HepG2 cells following SLC7A11 knockdown and overexpression. (**C**) HepG2 cell viability following SLC7A11 knockdown and overexpression. (**D**) Levels of hepatic injury- or ferroptosis-related proteins (COX-2, p21, ALOX12, HO-1, GPX4, and FTMT) in HepG2 cells following SLC7A11 knockdown and overexpression. (**E**-**G**) T-GSH, Fe^2+^, and MDA content in HepG2 cells following SLC7A11 knockdown and overexpression. ^*^P < 0.05, ^**^P < 0.01, ^***^P < 0.001

Next, the action of the downstream molecule, SLC7A11, on hepatic injury and ferroptosis were evaluated by “loss of function” and “gain of function” experiments. The knockdown efficiency of siSLC7A11 and overexpression efficiency of pcDNA3.1-SLC7A11 were verified by RT-qPCR and western blot assays (Fig. 6A and B). SLC7A11 knockdown resulted in a significant decrease in HepG2 cell viability, whereas SLC7A11 overexpression increased cell viability (Fig. 6C). SLC7A11 silencing promoted COX-2, p21, ALOX12 and HO-1 protein expression, while inhibited the expression of GPX4 and FTMT in HepG2 cells. And in contrary, the expression of COX-2, p21, ALOX12 and HO-1 protein expression were decreased in SLC7A11 over expression HepG2 cells, while the expression of GPX4 and FTMT were increased in SLC7A11 over expression HepG2 cells (Fig. 6D). Finally, we assessed the contents of ferroptosis biomarkers, including T-GSH, Fe^2+^, and MDA. These results show that ferroptosis was promoted following SLC7A11 knockdown, but suppressed in response to SLC7A11 overexpression (Fig. 6E-G). These outcomes indicate that SLC7A11 regulates hepatic injury and ferroptosis.

### Luteolin attenuates hepatic injury by inhibiting ferroptosis via SLC7A11

**Fig. 7:**
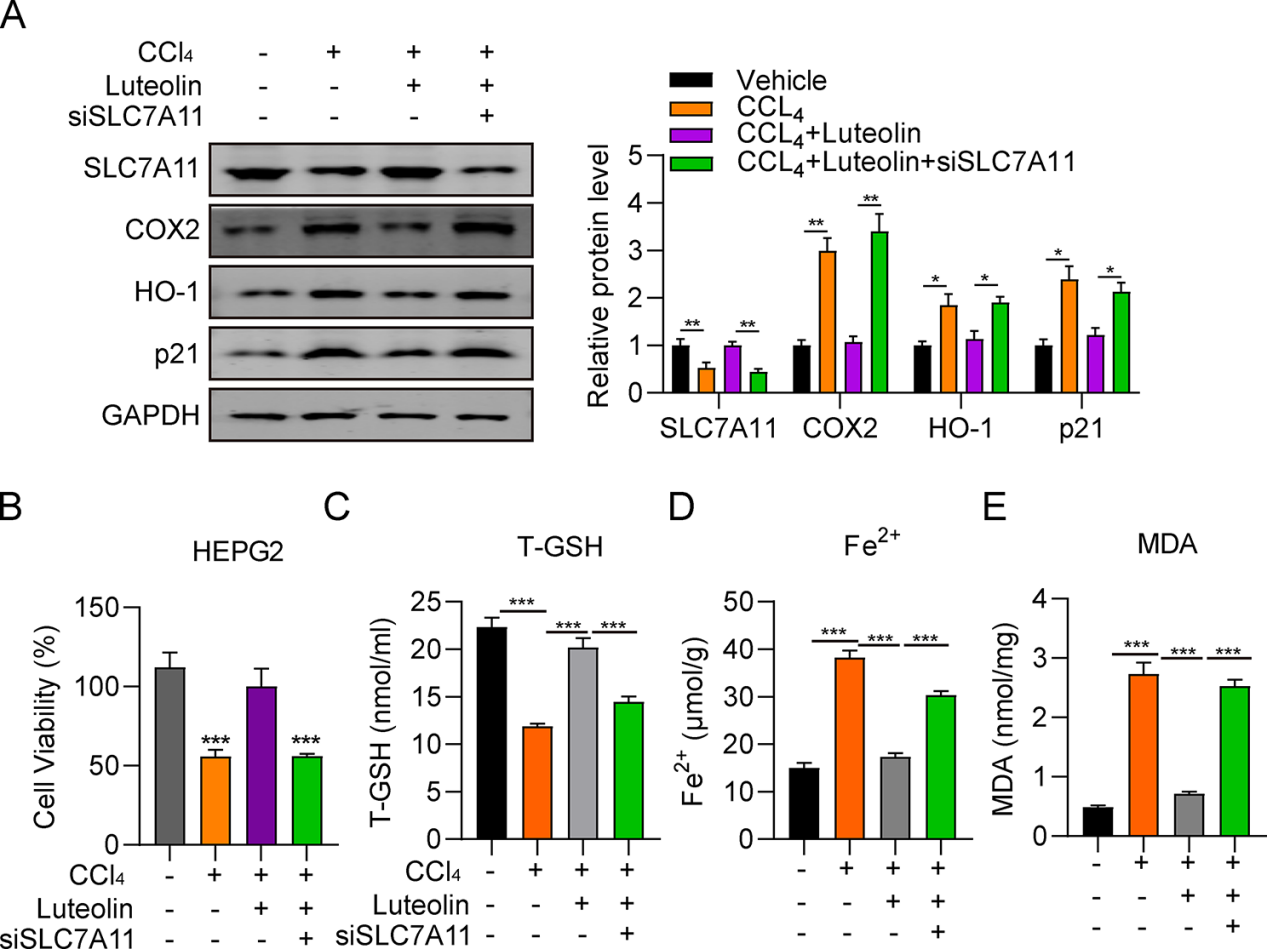
Luteolin attenuates hepatic injury by inhibiting ferroptosis via SLC7A11. (**A**) Comparison of SLC7A11, COX-2, HO-1, and p21 protein levels among HepG2 cells in vehicle, CCl_4_, CCl_4_ + luteolin, and CCl_4_ + luteolin + siSLC7A11 groups, determined by western blot. (**B**) Comparison of HepG2 cell viability among vehicle, CCl_4_, CCl_4_ + luteolin, and CCl_4_ + luteolin + siSLC7A11 groups, measured by CCK-8 assay. (**C**-**E**) Comparison of T-GSH, Fe^2+^, and MDA content among HepG2 cells in vehicle, CCl_4_, CCl_4_ + luteolin, and CCl_4_ + luteolin + siSLC7A11 groups, measured using corresponding kits. ^***^P < 0.001

We next investigated the effect of siSLC7A11 on luteolin attenuation of hepatic injury and ferroptosis. Western blot results indicated that SLC7A11 protein levels were greatly decreased in the CCl_4_ + luteolin + siSLC7A11 group relative to the CCl_4_ + luteolin group, indicating effective knockdown (Fig. 7A). Conversely, COX-2, HO-1, and p21 protein levels were all greatly elevated in the CCl_4_ + luteolin + siSLC7A11 group relative to the CCl_4_ + luteolin group, indicating that SLC7A11 was involved in the mechanism underlying luteolin attenuation of hepatic injury. Consistently, compared with the CCl_4_ + luteolin group, viability of HepG2 cells in the CCl_4_ + luteolin + siSLC7A11 group was significantly decreased (Fig. 7B). Additionally, a marked decrease of T-GSH levels and a substantial increase of Fe^2+^ and MDA levels were observed in the CCl_4_ + luteolin + siSLC7A11 group relative to the CCl_4_ + luteolin group (Fig. 7C–E). These results show that luteolin attenuates hepatic injury by inhibiting ferroptosis via SLC7A11, where SLC7A11 serves as a protector for hepatic injury and ferroptosis.

## Discussion

Recently, several studies have reported that luteolin attenuated hepatic Injury or liver fibrosis caused by CCl_4_. Domitrovic et al. showed that luteolin treatment greatly boosted metal content, up to control values for Mg and Cu, and even surpassing those of controls for Zn and Fe, in mice treated with CCl_4_ [25]. Further, they found that luteolin decreased oxidative stress induced by CCl_4_ [18]. The mechanism underlying the effects of luteolin on CCl_4_-induced liver fibrosis involves promotion of extracellular matrix degradation in fibrotic liver tissue [6]. Our findings show that luteolin administration can alleviate hepatic injury disruption of cell morphology and liver tissue structure in model mice treated with CCl_4_, and that serum ALT, AST, and ALP content were all significantly decreased, confirming previous reports that luteolin had a protective effect against hepatic injuries [7]. In addition, it is reported that ALOX12 is markedly upregulated in hepatic injury [26], and induction of ALOX12 facilitates lipid peroxide production in ferroptosis [27]. COX-2 is an essential enzyme in prostanoid biosynthesis, and has a protective role against hepatic injury [28]. As a cyclin-dependent kinase inhibitor, p21, is also involved in liver injury [29]. We studied the effects of luteolin on CCl_4_-treated hepatocellular HepG2 cells and found that it alleviated hepatic injury by improving cell viability, decreasing the expression of three hepatic injury related proteins (ALOX12, COX-2, and p21), and inhibiting apoptosis.

Ferroptosis is characterized by excessive production of lipid peroxides mediated by iron catalysis and is distinct from apoptosis, necroptosis, and pyroptosis [30]. Also, it occurs when intracellular glutathione peroxidase 4 (GPX4) is inhibited by a lowered GSH level [30]. Inhibition of GPX4 results in the accumulation of lipid peroxides, triggering cell membrane damage and eventually cell death in the absence of specific effector molecules [31]. Further, GPX4 is a common link between lipid homeostasis and ferroptosis [32]. FTMT has a critical role in iron homeostasis by attenuating cerebral ischemia/reperfusion injuries that result from ferroptosis inhibition [33]. Typical molecular features of ferroptosis include reduced GSH, and increased Fe^2+^ and lipid peroxidation levels [34]. MDA content serves as an indicator of lipid peroxidation [35]. We explored the effect of luteolin on ferroptosis by treating HepG2 cells with a series of luteolin concentrations (2.5–100 µM) and assessing cell viability, and found that 5 µM luteolin could increase cell viability. CCl_4_ treatment decreased GPX4 and FTMT protein levels, while luteolin treatment led to recovered expression of both proteins. CCl_4_ treatment also reduced T-GSH content and improved Fe^2+^ and MDA levels, whereas luteolin treatment had the opposite effect. These outcomes suggest that luteolin inhibits ferroptosis in hepatic cells. Furthermore, we used the ferroptosis inducer, erastin, to study whether luteolin inhibits ferroptosis in hepatic injury. Erastin can decrease intracellular GSH concentration by targeting the cystine/glutamate antiporter system, Xc- [30, 36]. Western blot, CCK-8, and flow cytometry assays confirmed our hypothesis. As a membrane-bound enzyme in liver macrophages, HO-1 hastens the death of erastin-induced ferroptotic cells [37], and HO-1 protein levels were increased in the CCl_4_ group, decreased by luteolin (CCl_4_ + luteolin group), and recovered on treatment with erastin (CCl_4_ + luteolin + erastin group).

Further, we used RNA-seq to search for key genes involved in luteolin attenuation of hepatic injury in HepG2 cells, and demonstrated that *SLC7A11* levels decreased following CCl_4_ treatment, while they increased after luteolin treatment, indicating that SLC7A11 has a protective effect against liver injuries. SLC7A11 is the light-chain subunit of a cystine/glutamate antiporter in the Xc- system; inhibition of the Xc- system results in decreased GSH levels, as well as ferroptosis initiation [12, 36]. SLC7A11 mediates antiporter activity of the Xc- system, while another subunit, SLC3A2, anchors SLC7A11 to the plasma membrane, thereby maintaining SLC7A11 protein stability [38]. It is established that SLC7A11 overexpression could suppress ferroptosis [38, 39]. SLC7A11 was involved in sorafenib triggers HSC ferroptosis, which attenuates liver injury and fibrosis [15].

We next investigated the role of SLC7A11 in hepatic injury and ferroptosis using “loss of function” and “gain of function” assays. SLC7A11 knockdown reduced cell viability, increased the expression of proteins involved in hepatic injury and ferroptosis, and decreased T-GSH level, as well as increased Fe^2+^ and MDA content. Conversely, ectopic SLC7A11 expression enhanced cell viability, reduced the expression of proteins that contribute to hepatic injury and ferroptosis, and increased T-GSH content, while decreased Fe^2+^ and MDA content. These results demonstrate that SLC7A11 regulates hepatic injury and ferroptosis. Based on our results, we hypothesized that luteolin attenuated hepatic injury and ferroptosis via SLC7A11. To test our hypothesis, we treated CCl_4_-induced HepG2 cells with a combination of luteolin and SLC7A11 knockdown, and observed that the downregulated COX-2, HO-1, and p21 proteins in luteolin-treated HepG2 cells were significantly increased in response to SLC7A11 knockdown, indicating that interfering with SLC7A11 expression promoted hepatic injury and ferroptosis. Similarly, HepG2 cell viability and T-GSH level recovery in response to luteolin were greatly decreased after SLC7A11 knockdown; that is, the decreases in Fe^2+^ and MDA content in response to luteolin were markedly increased after SLC7A11 knockdown.

## Conclusion

We report the first exploration of the relationships among hepatic injury, luteolin, and ferroptosis, and an investigation of the underlying molecular mechanism. Based on our findings, we conclude that luteolin inhibits hepatic injury by suppressing ferroptosis via regulating SLC7A11 expression and that SLC7A11 serves as a protector for hepatic injury, and is a potential alternative therapeutic target in hepatic injury.

### Electronic supplementary material

Below is the link to the electronic supplementary material.

Supplementary Material 1

Supplementary Material 2

## Data Availability

The datasets used and analyzed in this study are available from the corresponding author on reasonable request.

## Abbreviations

CCl4: Carbon tetrachloride
Luteolin: 3,4,5,7-tetrahydroxy flavone
GSH: glutathione
ALT: serum alanine aminotransferase
AST: aspartate aminotransferase
ALP: alkaline phosphatase
ALOX12: arachidonate 12-lipoxygenase
COX-2: cyclooxygenase-2
GPX4: glutathione peroxidase 4
FTMT: mitochondrial ferritin
HO-1: heme oxygenase 1
SLC7: A11 solute carrier family 7a member 11
PBST: phosphate buffered saline containing Tween
MDA: malondialdehyde
