# Supplementary figures and images for: Luteolin attenuates CCl4-induced hepatic injury by inhibiting ferroptosis via SLC7A11
Source: BMC Complement Med Ther. 2024 May 16;24:193. doi: 10.1186/s12906-024-04486-2 (PMC11100030; doi:10.1186/s12906-024-04486-2)

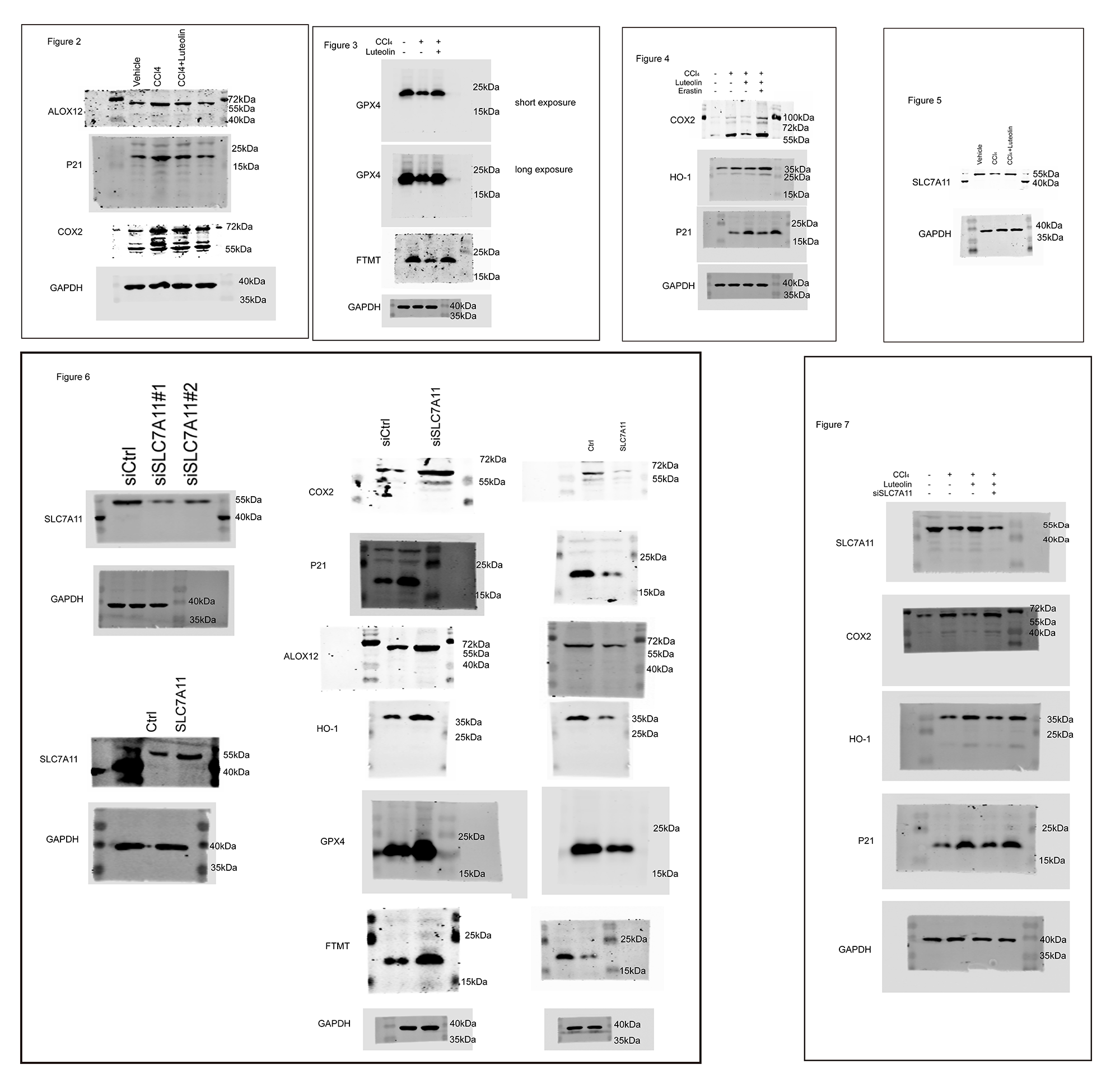

Supplement: Supplementary file 1 — Supplementary Material 1 [file 12906_2024_4486_MOESM1_ESM.tif]
