# Supplementary material for: Luteolin attenuates CCl4-induced hepatic injury by inhibiting ferroptosis via SLC7A11
Source: BMC Complement Med Ther. 2024 May 16;24:193. doi: 10.1186/s12906-024-04486-2 (PMC11100030; doi:10.1186/s12906-024-04486-2)
Supplement: Supplementary file 2 — Supplementary Material 2 [file 12906_2024_4486_MOESM2_ESM.docx]

**Supplementary Table 1. qPCR primers used in this study**

| Genes | Forward (5’-3’) | | Reverse (5’-3’) |
| --- | --- | --- | --- |
| *SLC7A11* | | TCTCCAAAGGAGGTTACCTGC | AGACTCCCCTCAGTAAAGTGAC |
| β-actin | | CATGTACGTTGCTATCCAGGC | CTCCTTAATGTCACGCACGAT |
